# Legacy benefits of blood pressure treatment on cardiovascular events are primarily mediated by improved blood pressure variability: the ASCOT trial

**DOI:** 10.1093/eurheartj/ehad814

**Published:** 2024-01-31

**Authors:** Ajay Gupta, William N Whiteley, Thomas Godec, Somayeh Rostamian, Cono Ariti, Judith Mackay, Andrew Whitehouse, Leila Janani, Neil R Poulter, Peter S Sever, the ASCOT-10 Investigators, Jehad Aldegather, David Collier, Christian Delles, Alexander Dyker, Mike Eaton, Simon Heller, David Hildick-Smith, Arni Kristinsson, Greg Lip, Graham MacGregor, Tom MacDonald, Ann Milward, Paul O’Hare, John Reckless, Carl Shakespeare, Soran Handrean, Adrian Stanley, Jacqueline Stokes, Simon Thom, John Webster

**Affiliations:** William Harvey Research Institute, Queen Mary University of London, UK; National Heart & Lung Institute, Imperial College London, Room 333, ICTEM Building, Du Cane Road, London W12 0NN, UK; Centre for Clinical Brain Sciences, University of Edinburgh, UK; William Harvey Research Institute, Queen Mary University of London, UK; National Heart & Lung Institute, Imperial College London, Room 333, ICTEM Building, Du Cane Road, London W12 0NN, UK; National Heart & Lung Institute, Imperial College London, Room 333, ICTEM Building, Du Cane Road, London W12 0NN, UK; National Heart & Lung Institute, Imperial College London, Room 333, ICTEM Building, Du Cane Road, London W12 0NN, UK; National Heart & Lung Institute, Imperial College London, Room 333, ICTEM Building, Du Cane Road, London W12 0NN, UK; Clinical Trials Unit, Imperial College London, UK; School of Public Health, Imperial College London, UK; National Heart & Lung Institute, Imperial College London, Room 333, ICTEM Building, Du Cane Road, London W12 0NN, UK

**Keywords:** ASCOT, Blood pressure variability, Cardiovascular events

## Abstract

**Background and Aims:**

Visit-to-visit systolic blood pressure variability (BPV) is an important predictor of cardiovascular (CV) outcomes. The long-term effect of a period of blood pressure (BP) control, but with differential BPV, is uncertain. Morbidity and mortality follow-up of UK participants in the Anglo-Scandinavian Cardiac Outcomes Trial-Blood Pressure-Lowering Arm has been extended for up to 21 years to determine the CV impact of mean systolic blood pressure (SBP) control and BPV during the trial, and amongst those allocated to amlodipine- and atenolol-based treatment.

**Methods:**

Eight thousand five hundred and eighty hypertensive participants (4305 assigned to amlodipine ± perindopril-based and 4275 to atenolol ± diuretic-based treatment during the in-trial period (median 5.5 years) were followed for up to 21 years (median 17.4 years), using linked hospital and mortality records. A subgroup of participants (*n* = 2156) was followed up 6 years after the trial closure with a self-administered questionnaire and a clinic visit. In-trial mean SBP and standard deviation of visit-to-visit SBP as a measure of BPV, were measured using >100 000 BP measurements. Cox proportional hazard models were used to estimate the risk [hazard ratios (HRs)], associated with (i) mean with SBP and BPV during the in-trial period, for the CV endpoints occurring after the end of the trial and (ii) randomly assigned treatment to events following randomization, for the first occurrence of pre-specified CV outcomes.

**Results:**

Using BP data from the in-trial period, in the post-trial period, although mean SBP was a predictor of CV outcomes {HR per 10 mmHg, 1.14 [95% confidence interval (CI) 1.10–1.17], *P* < .001}, systolic BPV independent of mean SBP was a strong predictor of CV events [HR per 5 mmHg 1.22 (95% CI 1.18–1.26), *P* < .001] and predicted events even in participants with well-controlled BP. During 21-year follow-up, those on amlodipine-based compared with atenolol-based in-trial treatment had significantly reduced risk of stroke [HR 0.82 (95% CI 0.72–0.93), *P* = .003], total CV events [HR 0.93 (95% CI 0.88–0.98), *P* = .008], total coronary events [HR 0.92 (95% CI 0.86–0.99), *P* = .024], and atrial fibrillation [HR 0.91 (95% CI 0.83–0.99), *P* = .030], with weaker evidence of a difference in CV mortality [HR 0.91 (95% CI 0.82–1.01), *P* = .073]. There was no significant difference in the incidence of non-fatal myocardial infarction and fatal coronary heart disease, heart failure, and all-cause mortality.

**Conclusions:**

Systolic BPV is a strong predictor of CV outcome, even in those with controlled SBP. The long-term benefits of amlodipine-based treatment compared with atenolol-based treatment in reducing CV events appear to be primarily mediated by an effect on systolic BPV during the trial period.

## Structured Graphical Abstract

**Structured Graphical Abstract:**
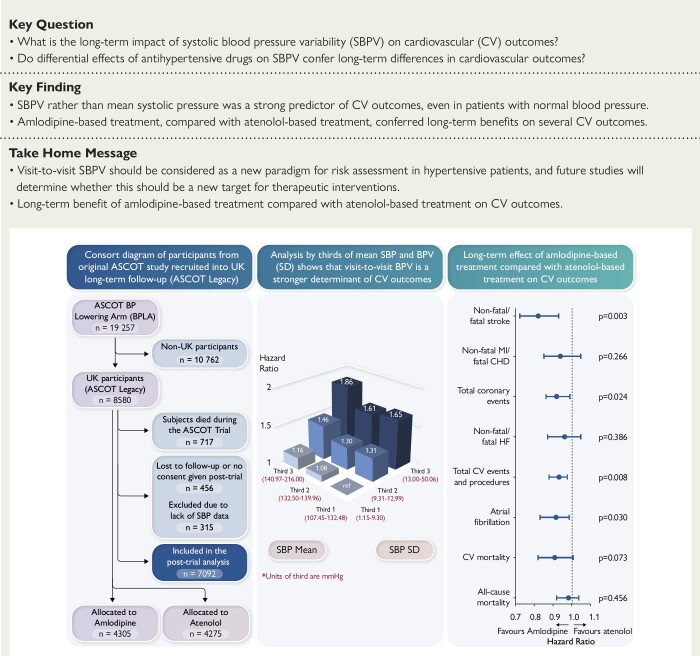
Blood pressure variability and blood pressure control in the 20-year follow-up of the ASCOT Legacy Study. BPV, blood pressure variability; SBP, systolic blood pressure; CHD, coronary heart disease; CV, cardiovascular; HF, heart failure; MI, myocardial infarction; SD, standard deviation.

**See the editorial comment for this article ‘Blood pressure variability: no longer a mASCOT for research nerds', by G. Parati**  ***et al*****., https://doi.org/10.1093/eurheartj/ehae023.**

## Introduction

Blood pressure variability (BPV) is an important predictor of morbidity and mortality based on observational studies.^1,2^ In the blood pressure (BP)-lowering arm (LA) of the Anglo-Scandinavian Cardiac Outcomes Trial-Blood Pressure-Lowering Arm (ASCOT-BPLA), an amlodipine-based treatment regimen reduced the incidence of cardiovascular (CV) outcomes and all-cause mortality compared with an atenolol-based regimen;^3^ but the small difference in mean BP between groups did not account for the observed differences in CV outcomes.^4^ Visit-to-visit systolic BPV was proposed as the determinant of the differences in outcomes between the amlodipine-based and atenolol-based treatment groups.^5^

After 16-year long-term follow-up to mortality outcomes (without information on non-fatal events), there was a persistent reduction in stroke deaths in those formerly assigned to amlodipine-based treatment.^6^ There were numerically fewer CV deaths, but the difference was not significant when compared with those assigned to atenolol-based treatment. We now report non-fatal events based on electronic health records of hospital admissions and further mortality outcomes up to a total of 21-year follow-up. In addition, we also report the findings from the ASCOT-10 sub-study in which we collected data on treatment, self-reported outcomes, and BP control in a quarter of these subjects after the end of the trial. These evaluations helped improve our understanding of what happened to these subjects after the trial closure. Recently, our observations on stroke and dementia outcomes have been published^7^ but with no attribution as to why the stroke protection existed beyond BP control. In the current analyses, we report the long-term impact of in-trial systolic BPV and mean BP on the long-term CV outcomes. We also report whether in the ASCOT legacy study the assignment to either of the original BP-lowering regimens conferred any long-term legacy benefits on fatal and non-fatal CV events or other new outcomes of interest, including the risk of atrial fibrillation and heart failure during the 21-year follow-up.

## Methods

The detailed ASCOT protocol, including study design, organization, clinical measurements, power calculations, recruitment rates, and baseline characteristics, has been published,^8^ and further information is available on the ASCOT website (http://www.ascotstudy.org.uk).

### ASCOT trial blood pressure arm and participant profile

Briefly, ASCOT was designed to compare two antihypertensive treatment strategies, amlodipine to which perindopril was added as necessary (amlodipine-based) and atenolol, to which bendroflumethiazide was added as necessary (atenolol-based)—the BPLA. This population consisted of hypertensive men and women, aged between 40 and 79 years at randomization, with at least three additional risk factors for CV disease, but with no history of prior coronary heart disease (CHD) events, currently treated angina, stroke, or transient ischaemic attack (TIA) within 3 months prior to randomization. The primary outcome was non-fatal myocardial infarction (MI) and fatal CHD. Patients were originally recruited between February 1998 and May 2000. In the Nordic countries, participants were recruited from 686 family practices and in the UK and Ireland, most participants were referred from family practices to the regional centres for the study. Blood pressure was measured throughout the trial, initially 6 weeks after randomization, at 3 months, 6 months, and 6 monthly thereafter until the end of the trial. Nineteen thousand two hundred and fifty-seven participants were randomized to the BPLA. The BPLA was prematurely stopped in December 2004 (at the recommendation of the Data Safety and Monitoring Board) on account of significantly higher mortality amongst those allocated to the atenolol-based treatment compared with those on amlodipine-based treatment.^3^

The study conformed to good clinical practice guidelines and the Declaration of Helsinki. The protocol and all subsequent amendments were reviewed and ratified by central and regional ethics review boards in the UK and by national ethics and statutory bodies in Ireland and the Nordic countries (Sweden, Denmark, Iceland, Norway, and Finland).

### ASCOT Legacy Cohort

**Figure 1:**
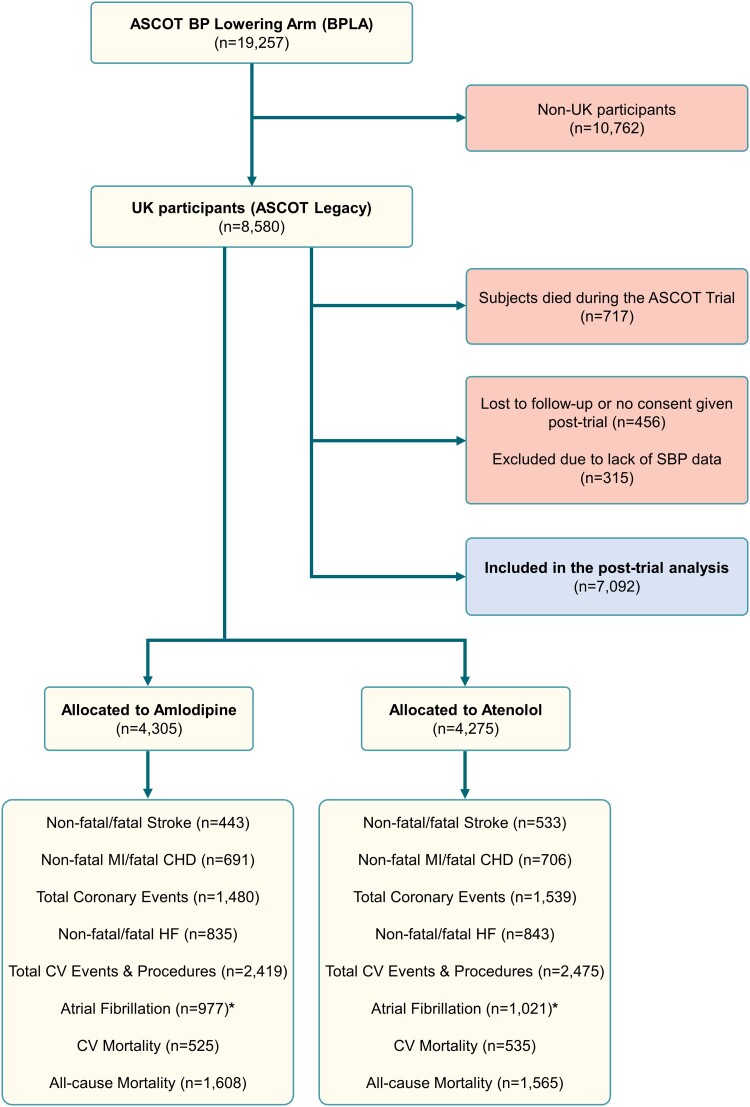
Diagram of BPLA of the ASCOT Legacy Cohort. *Subjects with prior atrial fibrillation at baseline were excluded from the analyses. Therefore, the denominators for the atrial fibrillation groups are different (amlodipine group: 4215 patients and atenolol group: 4245 patients were included)

Long-term follow-up of participants originally recruited in the Nordic countries was not possible using electronic records. Thus, 8580 participants from the UK, who were originally randomized to one of the two BP treatment regimens formed the ASCOT Legacy Cohort. All these participants were followed up until the end of the BPLA, during which period 717 of them died. Of the remainder, those who consented were followed up for death and hospitalization with NHS England and Public Health Scotland (*Figure 1*). In this report, we have used all post-trial reported deaths or hospitalizations before 31 January 2019. Overall, data on 7092 participants with completed records were analysed.

We used all available BP records during the trial visits (*n* = 100 933) over a 5-year in-trial period; a median of 13 visits per participant (three BP records per visit in most cases), after excluding those in the first 6 months after baseline, to estimate mean systolic blood pressure (SBP) (a measure of average BP control), and standard deviation (SD) of mean SBP across visits (an estimate of long-term visit-to-visit systolic BPV for each ASCOT Legacy subject).

In our previous publication,^1^ we carried out analyses using various measures of visit-to-visit BPV, including SD, coefficient of variation, and variation independent of the mean. The associations of BPV with CV outcomes were similar irrespective of the measure of variability used, so for the purpose of the current report, we restricted our analyses to the use of SD. We have also restricted our analyses to measurements based on SBP on account of the fact that in our previous publication,^1^ diastolic BP and diastolic BPV were much poorer predictors of CV outcomes.

A team of two physicians independently adjudicated all deaths, using pre-specified criteria that were consistent with the definitions used during the in-trial period. Outcomes for non-fatal events were obtained from electronic hospital records with previously validated code lists and classified them to the outcomes consistent with the original trial outcomes.

Our objectives were first, to determine the impact of in-trial systolic BPV and mean BP on the post-trial long-term CV outcome (a landmark analysis) and second, to compare the time to first occurrence of the following CV outcomes between those assigned to amlodipine-based and atenolol-based treatment: fatal and non-fatal stroke, non-fatal MI and fatal CHD, total coronary events, fatal and non-fatal heart failure, total CV events and procedures, atrial fibrillation, CV mortality, and all-cause mortality. Definitions for the various composite endpoints are available on http://www.ascotstudy.org.uk.

In 2011–12, after 6 years of trial closure and as a part of the ASCOT-10 study, a subgroup of surviving participants (*n* = 2156) was identified from 18 regional centres that had participated in the original trial in the UK and invited to complete a questionnaire on health and current medications. Of these, a further subgroup (*n* = 414) was recalled to the clinic for BP measurement and routine blood chemistry.

### Statistical methods

We analysed all UK participants in ASCOT. Where linkage was not possible (largely because participants had not consented to long-term mortality follow-up), participants were censored at the end of the trial follow-up period.

We assessed the association of both mean SBP level and systolic BPV estimated during the in-trial period with the first event of each long-term outcome after the trial closure beginning survival analysis from 5.5 years post-baseline (the median in-trial follow-up time) and including all patients who were alive at this time and who had at least three visits during the trial at which BP was recorded.

For each exposure, we conducted analyses both unadjusted and adjusted for a priori baseline confounders—age, sex, self-reported ethnicity, socioeconomic status (years of education), total cholesterol, smoking history, body mass index, and history of diabetes and the number of visits at which BP was measured, and the occurrence of stroke or MI historically or during the trial up to the post-trial period. For the analysis of systolic BPV, we further adjusted for mean SBP to get an estimate of the effect of systolic BPV independent of SBP level.

To assess the long-term outcomes of the original trial randomized treatments, we compared the first reported incidence of each endpoint between allocated antihypertensive treatment groups. Survival analyses were carried out using Cox proportional hazards models of time from baseline (randomization) to the first occurrence of fatal and non-fatal stroke, fatal and non-fatal CHD, total coronary events, fatal and non-fatal heart failure, total CV events, atrial fibrillation, CV mortality, and all-cause mortality. Analyses are reported unadjusted and adjusted for a priori confounders as above. For the analysis of atrial fibrillation, patients who were recorded as having atrial fibrillation at baseline were excluded.

We also carried out sensitivity analyses, in which we analysed time to these outcomes restricted to the post-trial follow-up period in the 7092 participants who were alive at the end of the trial.

For all analyses, censoring took place on death, withdrawal of consent, date of the end of linkage period, and out migration (participant moving away from the area).

For each analysis, we looked for evidence of effect modification by baseline age, sex, ethnicity, total cholesterol, body mass index, and diabetes. Stata 16 was used for all analyses.

### Ethical and other permissions

We obtained approval from the South East Scotland Research Ethics Committee (18/SS/0016), the Health Research Authority Confidentiality Advisory Group (18/CAG/0044), the Independent Group Advising on the Release of Data of NHS Digital, and the Public Benefit and Privacy Panel for Health and Social Care of NHS Scotland. We prepared this report with reference to the REporting of studies Conducted using Observational Routinely collected health Data (RECORD) Statement.^9^

## Results

In the ASCOT trial of 19 257 participants, 8580 participants were from England, Wales, or Scotland, of whom 7092 were alive and consented to further follow-up at the end of trial. Participants were followed up for a median of 17 years (interquartile range: 9–19) and a maximum of 21 years (*Figure 1*).

The 8580 UK participants were well balanced by allocated group (*Table 1*). On average, participants were 64 years (SD 8) at trial entry and the majority were male (81%) and had left education before 16 years of age (79%). At baseline, mean BP was 162/92 mmHg (SD 18/10). Participants had a history of diabetes (27%), stroke or TIA (12%), and other vascular disease (20%) (*Table 1*).

**Table 1:** Baseline characteristics at the time of treatment assignment of the ASCOT Legacy population

	ASCOT Legacy cohort		Post-trial analysis cohort
Values	Allocated to amlodipine (*n* = 4305)	Allocated to atenolol (*n* = 4275)	Total population (*n* = 7092)
Age (years), mean (SD)	64.2 (8.1)	64.2 (8.1)	63.8 (8.1)
Male sex, *n* (%)	3492 (81.1)	3468 (81.1)	5757 (81.2)
Female sex, *n* (%)	813 (18.9)	807 (18.9)	1335 (18.8)
Education (age left full time)			
12–16 years, *n* (%)	3437 (79.9)	3373 (78.9)	5565 (78.5)
≥17 years, *n* (%)	865 (20.1)	900 (21.1)	1527 (21.5)
Ethnicity, Whites, *n* (%)	3861 (89.7)	3840 (89.8)	6328 (89.2)
Current smoker, *n* (%)	1035 (24.0)	1006 (23.5)	1791 (25.3)
BMI (kg/m^2^), mean (SD)	28.9 (4.7)	28.9 (4.6)	28.9 (4.7)
SBP (mmHg), mean (SD)	162.1 (17.8)	161.6 (17.2)	161.4 (17.4)
DBP (mmHg), mean (SD)	92.3 (9.8)	91.9 (10.0)	92.2 (9.8)
Creatinine (mmol/L), mean (SD)	99.9 (17.4)	100.1 (17.8)	99.8 (17.0)
Total cholesterol (mmol/L), mean (SD)	5.9 (1.1)	5.9 (1.1)	5.9 (1.1)
HDL cholesterol (mmol/L), mean (SD)	1.3 (0.4)	1.3 (0.4)	1.3 (0.4)
LDL cholesterol (mmol/L), mean (SD)	3.8 (1.0)	3.8 (1.0)	3.8 (1.0)
Diabetes, *n* (%)	1139 (26.5)	1145 (26.8)	1838 (25.9)
Peripheral vascular disease, *n* (%)	359 (8.3)	383 (9.0)	583 (8.2)
Family h/o premature CHD, *n* (%)	734 (17.1)	745 (17.4)	1242 (17.5)
H/o previous stroke and TIA (>3 months ago), *n* (%)	507 (11.8)	492 (11.5)	775 (10.9)
Antihypertensive medication, *n* (%)	3961 (91.8)	3924 (91.8)	6500 (91.7)
Allocated to LL medication			
Atorvastatin, *n* (%)	1147 (26.6)	1170 (27.4)	1924 (27.1)
Placebo, *n* (%)	1152 (26.8)	1136 (26.6)	1886 (26.6)
Not part of LL arm, *n* (%)	2006 (46.6)	1969 (46.1)	3282 (46.3)

In BP analysis, data are missing for: 4 subjects for education, 171 subjects for creatinine, 424 subjects for LDL cholesterol.

*n*, number; CV, cardiovascular; CHD, coronary heart disease; SD, standard deviation; Y, year; BMI, body mass index; kg/m^2^, kilogram per square metres; SBP, systolic blood pressure; mmHg, millimetre of mercury; DBP, diastolic blood pressure; mmol/L, millimole/litter; TIA, transient ischaemic attack; LL, lipid-lowering; h/o, history of.

During the trial, differences in mean SBP between the two treatment arms were small [amlodipine-based 136.3 (SD 9.9) mmHg vs. atenolol-based 138.0 (SD 10.0) mmHg (*P* < .001)]. Differences in in-trial visit-to-visit systolic BPV were greater and significant [amlodipine-based 10.8 (SD 4.4), atenolol-based 12.8 (SD 4.8), *P* < .001].

In adjusted analyses, in-trial mean SBP was a significant predictor of post-trial long-term CV outcomes, including total CV events {hazard ratio (HR) 1.14 [95% confidence interval (CI) 1.10–1.17], *P* < .001}, total stroke [HR 1.19 (95% CI 1.11–1.26), *P* < .001], non-fatal MI and fatal CHD [HR 1.19 (95% CI 1.12–1.27), *P* < .001], and CV mortality [HR 1.22 (95% CI 1.16–1.29), *P* < .001], for each SD (10.0 mmHg) rise in mean SBP (*Table 2*). When further adjusted for in-trial mean SBP, the systolic BPV was a stronger predictor (*Table 3*), with HR of 1.22 (95% CI 1.18–1.26, *P* < .001) for total CV events, HR 1.20 (95% CI 1.11–1.29, *P* < .001) for total stroke, HR 1.25 (95% CI 1.16–1.35, *P* < .001) for non-fatal MI and fatal CHD, and HR 1.28 (95% CI 1.20–1.37, *P* < .001) for CV mortality per each SD of 5.0 mmHg rise in systolic BPV. Additional outcomes are reported in *Table 3*.

**Table 2:** The risk of cardiovascular and coronary heart disease events associated with each 10.0 mmHg of SD increase in mean systolic blood pressure in the post-trial analysis

Outcomes	*n* (%)	Crude HR (95% CI)	*P*-value	Adjusted^a^ HR (95% CI)	*P*-value
Non-fatal/fatal stroke	886 (12.5)	1.25 (1.18–1.32)	<.001	1.19 (1.11–1.26)	<.001
Non-fatal MI/fatal CHD	761 (10.7)	1.25 (1.17–1.393)	<.001	1.19 (1.12–1.27)	<.001
Total coronary events	2371 (33.4)	1.23 (1.19–1.28)	<.001	1.17 (1.13–1.22)	<.001
Non-fatal/fatal HF	1416 (20.0)	1.33 (1.27–1.39)	<.001	1.24 (1.18–1.30)	<.001
Total CV events and procedures	3934 (55.5)	1.20 (1.17–1.24)	<.001	1.14 (1.10–1.17)	<.001
Atrial fibrillation	1895 (26.7)	1.22 (1.17–1.27)	<.001	1.14 (1.09–1.90)	<.001
CV mortality	1060 (14.9)	1.31 (1.25–1.38)	<.001	1.22 (1.16–1.29)	<.001
All-cause mortality	3173 (44.7)	1.23 (1.19–1.27)	<.001	1.14 (1.11–1.18)	<.001

HR, hazard ratio; CI, confidence interval; MI, myocardial infarction; CHD, coronary heart disease; CV, cardiovascular; HF, heart failure.

^a^Adjusted for baseline age, sex, ethnicity, socioeconomic status (education), total cholesterol, body mass index, diabetes status, smoking habit, having had an MI or stroke prior to the post-trial analysis, and number of visits regarding BP measurements.

**Table 3:** The risk of cardiovascular and coronary heart disease events associated with each 5.0 mmHg of standard deviation rise in the blood pressure variability (visit-to-visit, standard deviation of mean systolic blood pressure) in the post-trial analysis

Outcomes	*n* (%)	Crude HR (95% CI)	*P*-value	Adjusted^a^ HR (95% CI)	*P*-value
Non-fatal/fatal stroke	886 (12.5)	1.38 (1.29–1.47)	<.001	1.20 (1.11–1.29)	<.001
Non-fatal MI/fatal CHD	761 (10.7)	1.36 (1.28–1.45)	<.001	1.25 (1.16–1.35)	<.001
Total coronary events	2371 (33.4)	1.38 (1.33–1.44)	<.001	1.25 (1.19–1.29)	<.001
Non-fatal/fatal HF	1416 (20.0)	1.47 (1.40–1.54)	<.001	1.25 (1.18–1.33)	<.001
Total CV events and procedures	3934 (55.5)	1.34 (1.30–1.38)	<.001	1.22 (1.18–1.26)	<.001
Atrial fibrillation	1895 (26.7)	1.37 (1.32–1.43)	<.001	1.26 (1.19–1.32)	<.001
CV mortality	1060 (14.9)	1.48 (1.40–1.56)	<.001	1.28 (1.20–1.37)	<.001
All-cause mortality	3173 (44.7)	1.37 (1.32–1.41)	<.001	1.21 (1.16–1.26)	<.001

HR, hazard ratio; CI, confidence interval; MI, myocardial infarction; CHD, coronary heart disease; CV, cardiovascular; HF, heart failure.

^a^Adjusted for baseline age, sex, ethnicity, socioeconomic status (education), total cholesterol, body mass index, diabetes status, smoking habit, having had an MI or stroke prior to the post-trial analysis, number of visits regarding BP measurements, and number of visits regarding BP measurements.

**Figure 2:**
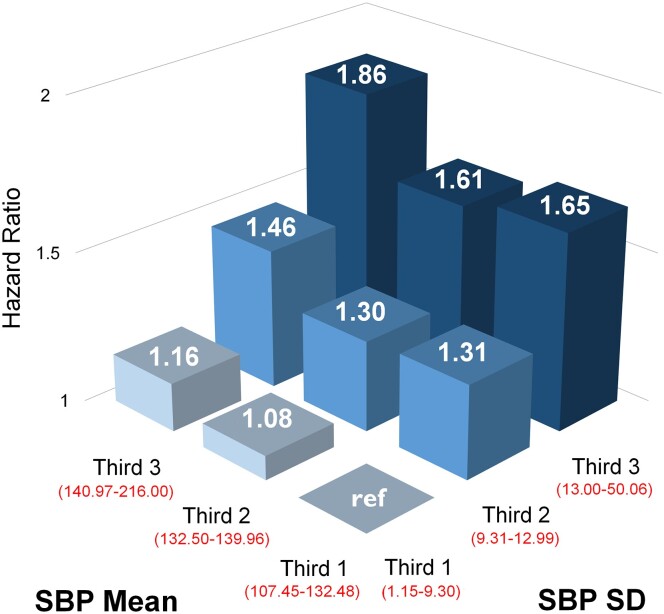
Risk of total cardiovascular events and procedures according to the mean systolic blood pressure and blood pressure variability in thirds. Association between thirds of mean systolic blood pressure and visit-to-visit systolic blood pressure variability (standard deviation of systolic blood pressure) with total cardiovascular events and procedures in the landmark analysis population. Range of mean systolic blood pressure: Third 1: 107.5–132.5 mmHg; Third 2: 132.5–139.9 mmHg; Third 3: 140.0–216.0 mmHg. Range of standard deviation for systolic blood pressure variability: Third 1: 1.15–9.30 mmHg; Third 2: 9.31–12.99 mmHg; Third 3: 13.00–50.06 mmHg

Analyses by thirds of mean SBP and systolic BPV supported findings that systolic BPV was a stronger determinant of CV outcomes than mean SBP (*Figure 2*, *Table 4* and Supplementary data online, *Figure S1*). In participants with a mean in-trial SBP of <140 mmHg, those in the highest third of BPV compared with the lowest third had an absolute increase in CV events of 16% over the 15-year post-trial follow-up (17 events per 1000 patient-years) (*Table 4*). Importantly, 53% of all CV events occurred in those whose in-trial BP was controlled to target levels advocated by contemporary guidelines (<140 mmHg SBP) and who would not have been considered for further treatment (*Table 4*). *Figure 2* shows that those with mean SBP below 133 mmHg throughout a period of 5 years have 31% and 65% excess risk of total CV events if the systolic BPV is between 9.3 to 13 and ≥13 mmHg, respectively. A formal test of interaction between mean BP and BPV was statistically significant (*P* < .001 unadjusted, *P* = .012 adjusted). These analyses provide strong evidence that the HRs differ across the various strata.

**Table 4:** Number and rate of total cardiovascular events and procedures according to the mean systolic blood pressure and blood pressure variability in thirds

	SBP variability (SD) (thirds)					
	1st (*n* = 2364) 1.15–9.30 mmHg		2nd (*n* = 2364) 9.30–13.00 mmHg		3rd (*n* = 2364) 13.00–50.06 mmHg	
	*n* (%)	Rate (per 1000 person-years)	*n* (%)	Rate (per 1000 person-years)	*n* (%)	Rate (per 1000 person-years)
**SBP mean (thirds)**						
1st (*n* = 2371) 107–133 mmHg	489/1107 (44.2)	29.3	445/816 (54.5)	38.9	276/448 (61.6)	48.3
2nd (*n* = 2358) 133–140 mmHg	412/862 (47.8)	32.6	460/838 (54. 9)	40.4	414/658 (62.9)	48.8
3rd (*n* = 2363) 140–216 mmHg	190/395 (48.1)	33.8	418/710 (58.9)	44.7	830/1258 (66.0)	55.9

SBP, systolic blood pressure; SD, standard deviation; *n*, number; mmHg, millimetre of mercury.

Whilst the treatment target in the original trial (<140/90) conformed to contemporary guidelines, a further analysis (data not shown) adopting a treatment target of <130/80 mmHg supported by some recent guidelines^10,11^ gave results similar to those shown in *Figure 2* for the lowest third of the SBP distribution.

**Figure 3:**
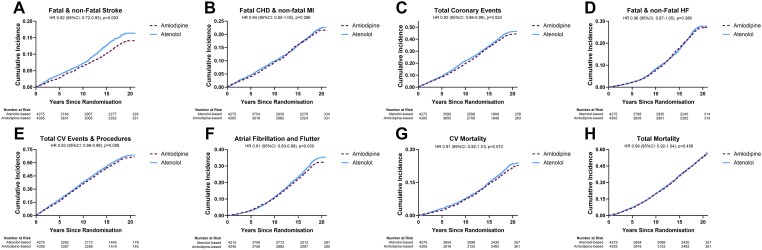
Kaplan–Meier curves showing the cumulative incidence of cardiovascular and coronary heart disease events amongst those allocated to amlodipine- and atenolol-based treatment in the ASCOT Legacy population. Kaplan–Meier curves show the association of amlodipine vs. atenolol allocation with (*A*) fatal and non-fatal stroke, (*B*) non-fatal myocardial infarction and fatal coronary heart disease, (*C*) total coronary events, (*D*) fatal and non-fatal heart failure, (*E*) total cardiovascular events and procedures, (*F*) new-onset of atrial fibrillation, (*G*) cardiovascular mortality, and (*H*) all-cause mortality in ASCOT Legacy population

*Table 5* shows the crude and adjusted HRs associated with the two treatment regimens over the 20-year follow-up. Those on amlodipine-based (vs. atenolol-based) treatment had a significantly reduced risk of stroke [HR 0.82 (95% CI 0.72–0.93), *P* = .003], total CV events and procedures [HR 0.93 (95% CI 0.88–0.98), *P* = .008], total coronary events [HR 0.92 (95% CI 0.86–0.99), *P* = .024], and atrial fibrillation [HR 0.91 (95% CI 0.83–0.99), *P* = .030]. There was weaker evidence for a difference in CV mortality [HR 0.91 (95% CI 0.82–1.01), *P* = .073]. However, there was no significant difference in the incidence of non-fatal MI and fatal CHD, heart failure or all-cause mortality (*Table 5*, *Figure 3*). For comparison, data are also shown for these outcomes in the original trial (see Supplementary data online, *Table S1*).

**Table 5:** The risk of cardiovascular and coronary heart disease events in those assigned to two treatment allocations in the ASCOT Legacy population

	Allocated to amlodipine-based treatment (*n* = 4305)		Allocated to atenolol-based treatment (*n* = 4275)					
Outcomes	*n* (%)	Rate (per 1000 person-years)	*n* (%)	Rate (per 1000 person-years)	Crude HR (95% CI)	*P*-value	Adjusted^a^ HR (95% CI)	*P*-value
Non-fatal/fatal stroke	443 (10.3)	7.54	522 (12.2)	8.88	0.83 (0.73–0.94)	.004	0.82 (0.72–0.93)	.003
Non-fatal MI/fatal CHD	691 (16.1)	11.78	706 (16.5)	12.04	0.96 (0.86–1.07)	.443	0.94 (0.85–1.05)	.266
Total coronary events	1480 (34.4)	27.66	1539 (36.0)	28.76	0.94 (0.87–1.01)	.069	0.92 (0.86–0.99)	.024
Non-fatal/fatal HF	835 (19.4)	14.27	843 (19.7)	14.41	0.98 (0.89–1.07)	.606	0.96 (0.87–1.05)	.386
Total CV events and procedures	2419 (56.2)	51.98	2475 (57.9)	53.19	0.94 (0.89–0.99)	.026	0.93 (0.88–0.98)	.008
Atrial fibrillation	977 (23.0)	17.59	1021 (24.2)	18.38	0.93 (0.85–1.01)	.085	0.91 (0.83–0.99)	.030
CV mortality	677 (15.5)	10.9	725 (17.0)	11.9	0.92 (0.83–1.02)	.115	0.91 (0.82–1.01)	.073
All-cause mortality	2025 (47.0)	32.7	2015 (47.1)	33.0	0.99 (0.93–1.05)	.729	0.98 (0.92–1.04)	.456

The number of subjects included in the analysis of these endpoints is 4305 for the amlodipine-based and 4275 for the atenolol-based arm, except for the outcome of atrial fibrillation where subjects with atrial fibrillation at baseline were excluded leaving 4245 in the amlodipine-based arm and 4215 in the atenolol-based arm.

*n*, number; PY, person years; HR, hazard ratio (risk on amlodipine vs risk on atenolol-based treatment); CI, confidence interval; MI, myocardial infarction; CHD, coronary heart disease; CV, cardiovascular; HF, heart failure.

^a^Adjusted for baseline age, sex, ethnicity, socioeconomic status (education), total cholesterol, body mass index, diabetes status, and smoking habit.

In sensitivity analyses, analysing time to these outcomes restricted to the post-trial follow-up period in the 7092 participants who were alive at the end of the trial and, who had at least 3 visits at which BP measurements were recorded, we found consistent results with the main analyses. Those on amlodipine-based (vs. atenolol-based) treatment had a significantly reduced risk of stroke [HR 0.84 (95% CI 0.73–0.98), *P* = .028], total CV events and procedures [HR 0.93 (95% CI 0.87–0.99), *P* = .022], total coronary events [HR 0.93 (95% CI 0.86–1.01), *P* = .078], and atrial fibrillation [HR 0.89 (95% CI 0.81–0.98), *P* = .015].

The subgroups of participants who were recalled during the follow-up period—a median of 6 years after the end of the trial—were broadly representative of the total ASCOT Legacy population (see Supplementary data online, *Table S2*). Blood pressure levels were similar 6 years post-trial closure amongst those formerly assigned amlodipine-compared with atenolol-based treatment (140.6/75.1 and 139.9/75.9 mmHg, respectively; Supplementary data online, *Table S3*). Supplementary data online, *Table S4* provides details of antihypertensive drug use in the participants formerly assigned to the two treatment groups. There was extensive treatment cross over between participants previously assigned amlodipine-based treatment to beta-blockers and/or diuretics. Likewise, in those formerly assigned atenolol-based treatment, many were now receiving calcium channel blockers and/or angiotensin-converting enzyme inhibitors or angiotensin receptor blockers.

## Discussion

In the companion paper to the original publication of the outcomes of ASCOT-BPLA,^4^ we concluded that the small differences in mean BP between the two treatment arms during the trial contributed minimally, if at all, to differences in CV outcomes. We subsequently reported that measurements of visit-to-visit SBP were powerful predictors of CV outcomes, including stroke and coronary events^1^ and that differences in the effect of the two treatment regimens on systolic BPV explained the benefits of the amlodipine-based treatment observed during the trial.^5^ The results obtained by longer term follow-up confirmed our findings at the end of the trial, although the differences in CV outcomes in those formerly assigned the two treatment regimens were less and likely explained by the impact of substantial crossover of treatments during the post-trial period.

In our current analyses, we have restricted our observations to those based on long-term visit-to-visit systolic BPV determined during the ASCOT trial. Our reasoning for this is that we previously reported the outcomes of alternative assessments of BPV in ASCOT, by short-term variability based on 24 h ambulatory BP monitoring and also within visit BPV. We clearly demonstrated that long-term BPV was a far better predictor of CV outcomes than alternative methods of BPV assessment^1^ and, therefore, used this measure in our current study.

Whilst the place of BPV in clinical practice is controversial, there is substantial evidence both from observational studies and clinical trials that long-term visit-to-visit BPV is an important determinant of CV outcomes, as reviewed by Stevens *et al*.^2^ The evidence base is compelling and has persuaded contemporary risk scores to incorporate a measure of visit-to-visit BPV in CV risk assessment^12^ and guidelines to highlight the importance of BPV in patient profiling.^11^

It is now evident from the long-term follow-up of ASCOT that, although BP control observed during the trial was a predictor of long-term CV outcomes, visit-to-visit systolic BPV was a stronger predictor of these events. We have also demonstrated that increased systolic BPV is an important determinant of CV outcome, even in those who, according to many guidelines, have controlled SBP to <140 mmHg. In these analyses, more than half of all long-term CV events occurred in this group of participants, who represented 44% of the trial population and who would hitherto not have been considered for additional therapeutic intervention. It is apparent that amongst those who have controlled BPs for a period of time (5 years), the systolic BPV of 13 mmHg or more is associated with significantly higher CV and CHD morbidity and mortality.

We previously reported that after 16 years of follow-up, on average 10.5 years after the closure of the trial, there was a persistent reduction in stroke mortality in favour of those participants formerly assigned amlodipine-based treatment compared with atenolol-based treatment.^6^ In the current analyses, we have extended these observations on subjects who have been followed up for up to 21 years and incorporated extensive findings from electronic records of hospital admissions for CV events together with accumulated data on mortality.

A persistent benefit on stroke, all coronary events, and all CV events were seen in those patients formerly assigned to amlodipine-based treatment. These proportional reductions in events were slightly less than those recorded during the original trial and probably accounted for by the extensive crossover of treatments in the post-trial period between the two formerly randomized groups. There was also a reduction in the incidence of atrial fibrillation associated with amlodipine-based treatment which, together with the reduction in BP variability, were likely explanations for the persistence of beneficial effects on the risk of stroke (*Structured Graphical Abstract*).

Several other studies have confirmed the differential effects of antihypertensive drugs on BPV.^13,14^ Long-acting dihydropyridine calcium channel blockers have consistently been shown to lower visit-to-visit BPV. Whilst the underlying mechanisms remain speculative, it is likely that the combined haemodynamic effect resulting from intense peripheral precapillary vasodilatation together with increased large and small arterial vascular compliance play a contributory role.^15^ Thiazide and thiazide-like diuretics also have a modest effect reducing visit-to-visit BPV. Whilst angiotensin-converting enzyme inhibitors and angiotensin receptor blockers have little or no effect on BPV, the evidence that beta-blockers such as atenolol increase BPV is fairly consistent.^13^ It is possible that the lack of protection of atenolol against stroke reported in some studies is not only due to its effect on BPV but also to the fact that the increased wave reflection associated with beta-blocker-induced bradycardia, increases end SBP, and augmentation index. Whether third generation beta-blockers, with or without vasodilating properties, would share this increased risk of stroke is not known and would require further outcome studies.

So-called legacy effects of former treatment have most commonly been reported in trials of lipid-lowering with statins^6,16^ and variously attributed to off-target effects of the drugs. However, the phenomenon has also been reported in some trials of antihypertensive drugs^17^ but not confirmed in follow-up of the SPRINT trial.^18^ Legacy effects were also reported in the UKPDS trial of an oral hypoglycaemic agent;^19^ thus, the phenomenon appears to be more generalized and may not be due to specific effects of individual drugs. In most of these long-term follow-up studies differences in cholesterol, blood sugar, and BP, evident in the trials and accounting for the differences in-trial outcomes, were minimized in the post-trial period, so could not account for the long-term benefits. It is possible that target organ benefits occurring during the trials resulting from risk factor reduction could confer longer term advantages. Finally, the ‘catch up’ hypothesis (Kausik Ray, personal communication) has been invoked to explain this phenomenon—simply that those assigned to the less effective treatment during the trial, fail to catch up with those assigned to more effective treatment despite the fact that similar treatments may be made available to all subjects during long-term follow-up.

In the current study, we had the advantage of recall of subgroups of participants during the long-term follow-up. Information derived from these visits demonstrated that, in addition to the fact that BP levels were similar, there was a much greater overlap of drug treatment between the two arms of the trial than reported during the trial. More subjects received amlodipine and angiotensin-converting enzyme inhibitors in those formerly assigned atenolol-based treatment, and the latter were more likely to receive either amlodipine or angiotensin-converting enzyme inhibitors. Thus, whatever the explanation for the benefits of amlodipine-based treatment reported in the original trial, such factors could have persisted, although potentially diluted, during the long-term follow-up.

We have no measure of BPV during follow-up, but if treatment favoured amlodipine-based treatment in those formerly assigned amlodipine, then this could account, at least in part, for the beneficial effects reported in this article. Importantly, few antihypertensive classes of drugs have a substantial effect on visit-to-visit BPV, which seems to be largely accounted for by alterations in compliance and structure in large arteries.^20^ In comparisons between drugs, long-acting calcium channel blockers, such as amlodipine and, to a lesser extent non-loop diuretics, reduce long-term visit-to-visit BPV compared with other classes of drugs.^13^

Given the observations that a substantial number of events occur in subjects with normal or controlled BP but high BPV, an important question is to what extent might long-acting calcium channel blockers such as amlodipine reduce the risk of CV events in this group of patients. In the original ASCOT trial, an amlodipine-based treatment regimen reduced the incidence of stroke events by 22% and coronary events by 15% compared with atenolol-based treatment.^3^ Subsequent *post hoc* analyses showed that this was best explained by the reduction of ∼15% in visit-to-visit systolic BPV.^5^ This reduction in CV events was independent of mean SBP. This therefore provides an estimate of the potential benefits of amlodipine-based treatment in subjects with well-controlled SBP but high BPV, however, a definitive answer to this question requires a prospectively designed randomized controlled clinical trial.

Differences in atrial fibrillation were not reported during the original trial, but during long-term follow-up there were significantly fewer cases recorded amongst those originally assigned amlodipine-based treatment. Given the strong association between atrial fibrillation and the incidence of stroke, it is possible that this could contribute to the difference in stroke outcomes observed during the long-term follow-up.

There are limitations to our analyses. We have limited BP measurement and BP treatment data from the sub-study samples post-trial. However, these are more likely than not to be representative of the trial population. Both in the original trial and from post-trial evaluation, we only have data on prescribed medications; also, non-fatal outcomes using (ICD-10 codes) have their limitations. However, the adjudicated data on mortality support the results of assessment of combined fatal and non-fatal outcomes.

## Conclusions

Contemporary clinical practice, based on current guidelines, dictates that treatment decisions in hypertensive patients are determined by levels of SBP and diastolic BP. Our studies, however, provide robust evidence that visit-to-visit BPV is a far more powerful determinant of CV outcome and that at least half of all CV events in our cohort occurred in those with controlled BP but high BPV. We suggest that cut-off of SD for systolic BPV of 13 or more amongst those with ‘controlled BPs for a period of time’ may in itself suggest a need to act on the excess risk, although we acknowledge, this needs to be supported by further research to establish BPV as a new paradigm for the determinant of thresholds and targets for intervention.

This study also confirms the long-term benefits of amlodipine-based treatment in reducing the risk of stroke, coronary events, and all CV events. The significant reduction in incident stroke beyond the end of the trial may be mediated in part by the reduced incidence of atrial fibrillation and lower SBP variability associated with amlodipine-based treatment.

Long-term benefits of particular treatment strategies have profound clinical implications. The original results of ASCOT-BPLA influenced national and international guidelines for hypertension.^21,22^ The current observations extend the benefits reported in the trial for the amlodipine-based treatment regimen and highlight the increasing importance of visit-to-visit systolic BPV in the prediction of long-term CV outcomes.

## Supplementary Material

ehad814_Supplementary_Data

## Data Availability

Investigators wishing to access these data need to contract with NHS Digital and NHS Scotland, obtain the relevant ethical and data governance permissions, and have an analysis environment compliant with the Data Security and Protection Toolkit (www.dsptoolkit.nhs.uk). Other data from this study (code lists, statistical code) are available. Tabular data can be shared with collaborators if costs for further data extraction and analyses can be covered, by application to the ASCOT chief investigator, P.S.S.

## Pre-registered Clinical Trial Number

NCT 01499511.
