# Supplementary material for: Legacy benefits of blood pressure treatment on cardiovascular events are primarily mediated by improved blood pressure variability: the ASCOT trial
Source: Eur Heart J. 2024 Jan 31;45(13):1159–69. doi: 10.1093/eurheartj/ehad814 (PMC10984564; doi:10.1093/eurheartj/ehad814)
Supplement: ehad814_Supplementary_Data [file ehad814_supplementary_data.zip › Sup Tables 1 - 4 + Sup plus Fig Leg_PL.docx]

**SUPPLEMENTARY MATERIALS**

**Legacy benefits of blood pressure treatment on cardiovascular events are primarily mediated by improved blood pressure variability: the ASCOT trial**

Short Title: Blood Pressure Variability and Blood Pressure Control in the ASCOT Legacy Study

**Authors:** Ajay Gupta^1,2^, William N Whiteley^3^, Thomas Godec^1^, Somayeh Rostamian^2^, Cono Ariti^2^ ,Judith Mackay^2^, Andrew Whitehouse^2^, Leila Janani^5^, Neil R Poulter^4^, Peter S Sever^2^, and the ASCOT-10 Investigators

|  | **Allocated to amlodipine**  **(n=4,305)** | **Allocated to atenolol**  **(n=4,275)** |  |  |
| --- | --- | --- | --- | --- |
| **Outcomes** | **n (%)** | **n (%)** | **Crude HR** | **P-value** |
| Non-fatal/fatal Stroke | 137 (3.2%) | 158 (3.7%) | 0.86 (0.68-1.08) | 0.182 |
| Non-fatal MI/fatal CHD | 178 (4.1%) | 195 (4.6%) | 0.90 (0.74-1.11) | 0.326 |
| Total Coronary Events | 340 (7.9%) | 360 (8.4%) | 0.93 (0.80-1.08) | 0.358 |
| Non-fatal/fatal HF | 56 (1.3%) | 67 (1.6%) | 0.83 (0.58-1.18) | 0.291 |
| Total CV Events & Procedures | 600 (13.9%) | 637 (14.9%) | 0.93 (0.83-1.04) | 0.182 |
| Atrial Fibrillation | 166 (3.9%) | 139 (3.3%) | 0.92 (0.84-1.02) | 0.070 |
| CV Mortality | 114 (2.6%) | 149 (3.5%) | 0.76 (0.59-0.97) | 0.026 |
| All-cause Mortality | 347 (8.1%) | 371 (8.7%) | 0.93 (0.80-1.07) | 0.295 |

**Table S1. Numbers (and proportions) of those who have had CV and CHD outcomes (first event only) during the in-trial period**

**Abbreviations:** n: number, MI: Myocardial Infarction, CHD: Coronary Heart Disease; CV: Cardiovascular, HF: Heart Failure.

* Subjects with prior atrial fibrillation (AF) at baseline were excluded from the analyses. Therefore, the denominators for the AF groups are different (Amlodipine group: 4,215 patients and Atenolol group: 4,245 patients were included)

**Table S2. Baseline characteristics amongst those participating in the post-trial evaluation stratified by the treatment allocation: the ASCOT-10 population**

| **Values** | **Total Population**  **(n=2,156)** | **Allocated to amlodipine**  **(n=1,117)** | **Allocated to atenolol**  **(n=1,039)** |
| --- | --- | --- | --- |
| Age, mean (SD) | 61.7 (7.2) | 61.9 (7.2) | 61.5 (7.2) |
| Male, n (%) | 1,828 (84.8%) | 934 (83.6%) | 894 (86.0%) |
| Female, n (%) | 328 (15.2%) | 183 (16.4%) | 145 (14.0%) |
| Education (year), n (%) |  |  |  |
| 12-16 Y | 1,530 (71.0%) | 815 (73.0%) | 715 (68.8%) |
| 17+ Y | 626 (29.0%) | 302 (27.0%) | 324 (31.2%) |
| Ethnicity, White, n (%) | 1,937 (89.8%) | 1,007 (90.2%) | 930 (89.5%) |
| Current smokers, n (%) | 491 (22.8%) | 259 (23.2%) | 232 (22.3%) |
| BMI (kg/m2), mean (SD) | 29.2 (4.6) | 29.2 (4.7) | 29.2 (4.5) |
| BMI Groups |  |  |  |
| BMI: ≤24.9. n (%) | 355 (16.5%) | 182 (16.3%) | 173 (16.7%) |
| BMI: 25-29.9, n (%) | 1,009 (46.8%) | 525 (47.0%) | 484 (46.6%) |
| BMI: 30-34.9 n (%) | 577 (26.8%) | 300 (26.9%) | 277 (26.7%) |
| BMI: ≥35, n (%) | 214 (9.9%) | 110 (9.8%) | 104 (10.0%) |
| SBP (mmHg), mean (SD) | 158.6 (16.2) | 159.1 (16.7) | 158.2 (15.7) |
| DBP (mmHg), mean (SD) | 92.8 (9.3) | 92.8 (9.4) | 92.7 (9.2) |
| Creatinine (mmol/l), mean (SD) | 99.0 (15.4) | 98.9 (15.7) | 99.1 (15.1) |
| Total cholesterol (mmol/l), mean (SD) | 5.9 (1.1) | 6.0 (1.1) | 5.9 (1.0) |
| LDL cholesterol (mmol/l), mean (SD) | 3.8 (0.9) | 3.8 (0.9) | 3.8 (0.9) |
| HDL cholesterol (mmol/l), mean (SD) | 1.3 (0.3) | 1.3 (0.3) | 1.3 (0.3) |
| Diabetes, n (%) | 503 (23.3%) | 269 (24.1%) | 234 (22.5%) |
| Peripheral vascular disease, n (%) | 89 (4.1%) | 44 (3.9%) | 45 (4.3%) |
| Family h/o premature CHD, n (%) | 431 (20.0%) | 240 (21.5%) | 191 (18.4%) |
| H/o previous stroke and TIA (> 3 months ago), n (%) | 163 (7.6%) | 88 (7.9%) | 75 (7.2%) |
| Antihypertensive medication, n (%) | 1,976 (91.7%) | 1,028 (92.0%) | 948 (91.2%) |
| Allocated to LL medication, n (%) |  |  |  |
| Atorvastatin | 596 (27.6%) | 292 (26.1%) | 304 (29.2%) |
| Placebo | 601 (27.9%) | 323 (29.0%) | 278 (26.8%) |

**Abbreviations:** n: number, CV: cardiovascular, CHD: coronary heart disease; SD: standard deviation, Y: year, BMI: body mass index, kg/m2: kilogram per square meters, SBP: systolic blood pressure, mmHg: millimetre of mercury, DBP: diastolic blood pressure, LDL: low-density lipoproteins, mmol/l: millimole/litter, HDL: high-density lipoproteins, TIA: transient ischemic attack, LL: lipid-lowering.

**Table S3. Results of the physical evaluation after six years from the end of trial in the ASCOT-10 population**

|  | **Total Population*****  (n=414) | **Allocated to amlodipine**  (n=214) | **Allocated to atenolol**  (n=200) |
| --- | --- | --- | --- |
| SBP (mmHg), mean (SD) | 140.3 (17.7) | 140.6 (18.7) | 139.9 (16.4) |
| DBP (mmHg), mean (SD) | 75.5 (10.61) | 75.1 (11.2) | 75.9 (9.9) |
| Total Cholesterol (mmol/l), mean (SD) | 3.9 (0.9) | 4.0 (1.0) | 3.9 (0.9) |
| LDL (mmol/l), mean (SD) | 2.1 (0.7) | 2.2 (0.8) | 2.1 (0.7) |
| HDL (mmol/l), mean (SD) | 1.4 (0.4) | 1.4 (0.4) | 1.3 (0.4) |
| Fasting Glucose (mmol/l), mean (SD) | 6.1 (1.9) | 6.0 (1.8) | 6.1 (2.0) |

**Abbreviations:** SBP: systolic blood pressure, mmHg: millimetre of mercury, DBP: diastolic blood pressure, LDL: low-density lipoproteins, mmol/l: millimole/litter, HDL: high-density lipoproteins.

*** Please note that approximately 414 patients were tested for blood measurements.

**Table S4. Anti-hypertensive treatment prescription in the community amongst those who were evaluated post-trial in the ASCOT-10 Study**

| **ASCOT-10** | **Medications** | **Allocated to amlodipine-based treatment during the trial** | **Allocated to atenolol-based treatment during the trial** |
| --- | --- | --- | --- |
| Number of participants |  | 1,117 | 1,039 |
| Post-trial antihypertensive medications (% of participants) | ACEi/ARBs | 861 (77.1) | 680 (65.4) |
|  | Beta-blockers | 239 (21.4) | 357 (34.4) |
|  | CCBs | 751 (67.2) | 474 (45.6) |
|  | Diuretics | 326 (29.2) | 507 (48.8) |
|  | Others* | 467 (41.8) | 496 (47.7) |
| Number of antihypertensive medications each patient used** | 1 | 168 (15.0) | 149 (14.3) |
|  | 2 | 402 (36.0) | 369 (35.5) |
|  | ≥3 | 474 (42.4) | 453 (43.6) |
| Post-trial antihypertensive medication combinations. | ACEi/ARBs + CCBs | 582 (52.1) | 322 (31.0) |
|  | ACEi/ARBs + Beta-blockers | 185 (16.6) | 202 (19.4) |
|  | Beta-blockers + Diuretics | 85 (7.6) | 162 (15.6) |
|  | ACEi/ARBs + Diuretics | 248 (22.2) | 318 (30.6) |
|  | Beta-blockers + CCBs | 138 (12.4) | 127 (12.2) |
|  | CCBs + Diuretics | 174 (15.6) | 200 (19.2) |

**Abbreviations:** n: number. ACEi: Angiotensin-converting enzyme inhibitors (ACE inhibitors); ARBs: and angiotensin receptor blockers, CCBs: Calcium channel blockers

*Includes alpha-blockers and clonidine-like drugs

**In each group, 6.5% of participants did not report their medication

**Figure Legend**

**Figure S1. The risk of CV and CHD events associated with each third of cumulative mean SBP and visit-to-visit SBP variability in the landmark analysis population**

Association between thirds of mean systolic blood pressure (SBP) and visit-to-visit systolic blood pressure variability (SD) with **A.** Fatal and non-fatal stroke, **B.** Non-fatal myocardial infarction and fatal coronary heart disease, **C.** Total coronary events, **D.** Fatal and non-fatal heart failure, **E.** Total cardiovascular events and procedures, **F.** New-onset of atrial fibrillation, **G.** CV mortality, and **H**. All-cause mortality in ASCOT Legacy population.

Range of mean SBP: Third 1: 107.5 to132.5 mmHg; Third 2: 132.5 to139.9mm Hg; Third 3: 140.0 to 216.0 mmHg.

Range of SD for systolic BP variability: Third 1: 1.15 to 9.30 mmHg; Third 2: 9.31 to 12.99 mmHg; Third 3: 13.00 to 50.06 mmHg.
