# Supplementary figures and images for: Legacy benefits of blood pressure treatment on cardiovascular events are primarily mediated by improved blood pressure variability: the ASCOT trial
Source: Eur Heart J. 2024 Jan 31;45(13):1159–69. doi: 10.1093/eurheartj/ehad814 (PMC10984564; doi:10.1093/eurheartj/ehad814)

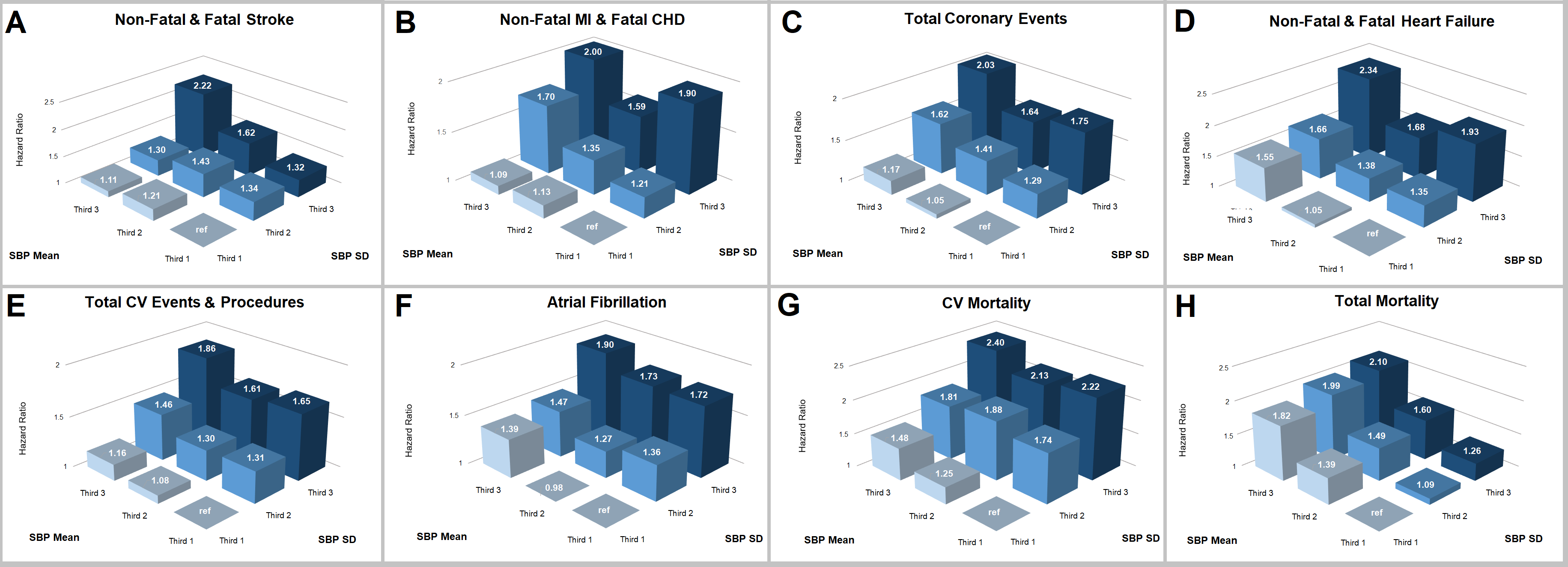

Supplement: ehad814_Supplementary_Data [file ehad814_supplementary_data.zip › SUP FIGURE 1 (003).png]
